# Birth prevalence for congenital limb defects in the northern Netherlands: a 30-year population-based study

**DOI:** 10.1186/1471-2474-14-323

**Published:** 2013-11-16

**Authors:** Ecaterina Vasluian, Corry K van der Sluis, Anthonie J van Essen, Jorieke E H Bergman, Pieter U Dijkstra, Heleen A Reinders-Messelink, Hermien E K de Walle

**Affiliations:** 1 Department of Rehabilitation Medicine, University Medical Centre Groningen, University of Groningen, Groningen, The Netherlands; 2 Department of Genetics, University Medical Centre Groningen, University of Groningen, Groningen, The Netherlands; 3 Department of Genetics, EUROCAT Registration of Congenital Anomalies, University Medical Centre Groningen, University of Groningen, Groningen, The Netherlands; 4 Department of Oral and Maxillofacial Surgery, University of Groningen, University Medical Centre Groningen, Groningen, The Netherlands; 5 Rehabilitation Center ‘Revalidatie Friesland’, Beetsterzwaag, The Netherlands

**Keywords:** Congenital limb deformities, Congenital abnormalities, Prevalence, Epidemiology

## Abstract

**Background:**

Reported birth prevalences of congenital limb defects (CLD) vary between countries: from 13/10,000 in Finland for the period 1964–1977 to 30.4/10,000 births in Scotland from 1964–1968. Epidemiological studies permit the timely detection of trends in CLD and of associations with other birth defects. The aim of this study is to describe the birth prevalence of CLD in the northern Netherlands.

**Methods:**

In a population-based, epidemiological study we investigated the birth prevalences of CLD for 1981–2010. Data were collected by the European Surveillance of Congenital Anomalies in the northern Netherlands (EUROCAT-NNL). We excluded malpositions, club foot, and dislocation/dysplasia of hips or knees. Trends were analysed for the 19-year period 1992–2010 using χ^2^ tests, as well as CLD association with anomalies affecting other organs.

**Results:**

The birth prevalence of CLD was 21.1/10,000 births for 1981–2010. There was an overall decrease in non-syndromic limb defects (*P* = 0.023) caused by a decrease in the prevalence of non-syndromic syndactyly (*P* < 0.01) in 1992–2010. Of 1,048 children with CLD, 55% were males, 57% had isolated defects, 13% had multiple congenital anomalies (MCA), and 30% had a recognised syndrome. The upper:lower limb ratio was 2:1, and the left:right side ratio was 1.2:1. Cardiovascular and urinary tract anomalies were common in combination with CLD (37% and 25% of cases with MCA). Digestive-tract anomalies were significantly associated with CLD (*P* = 0.016).

**Conclusions:**

The birth prevalence of CLD in the northern Netherlands was 21.1/10,000 births. The birth prevalence of non-syndromic syndactyly dropped from 5.2/10,000 to 1.1/10,000 in 1992–2010.

## Background

Limb defects seen in childhood are mainly congenital and occur when a part of or the entire limb fails to form normally during pregnancy. Reduction defects may be disabling limb defects due to the failure of several elements to form properly [1]. Less disabling limb defects are polydactyly, defined as complete or partial supernumerary digits, and syndactyly, fusion of two or more digits [2]. Disruptive events appear to be the most common cause of congenital limb defects (CLD) [3]. During the gestational period, disruptive events, such as amniotic band or vascular disruptions, may cause amputation or hypoperfusion of the developing limbs [4]. Various CLD are due to prenatal exposure to different teratogens [5], the best-known example of which is thalidomide, which caused a wide range of CLD, especially intercalary reductions and preaxial defects, in the 1960s [6]. To prevent further tragedies, several international registries of congenital defects were established. The European Surveillance of Congenital Anomalies (EUROCAT) network of registries in thirty-seven countries and the International Clearinghouse for Birth Defects Surveillance and Research are two such registries that have the goal of monitoring birth defects [7,8]. Monitoring CLD birth prevalences (BP) permits estimates of how common CLD are in the general population, early detection of risk factors for CLD and its associations with other congenital anomalies, and comparison of standard data collections.

Complete epidemiological descriptions of all CLD in different countries are rather scarce. In Finland a BP of 13/10,000 births was found for the period 1964–1977, whereas in Scotland it was estimated at 30/10,000 for 1964–1968 [9,10]. More common reports in the literature are studies on specific types of CLD, especially of congenital reduction defects [11-22]. Reported BPs of reduction defects vary widely in time, and between countries. In Italy the prevalence was as low as 4.8/10,000, while in France it was 10.4/10,000 from 1979–1987 [13,23,24]. The BP/10,000 of CLD in the Netherlands from 1997–2007 was 9.9 for polydactyly, 7.0 for syndactyly and 1.4 for reduction defects [25].

There is no up-to-date, detailed information on CLD in the Dutch population available. Therefore, the aim of this study is to describe the epidemiology of CLD in a population-based study in the northern Netherlands for the period 1981–2010.

## Methods

### Data source

The CLD cases have been collected by EUROCAT in the northern Netherlands (EUROCAT-NNL) since 1981. Children or foetuses with one or more major congenital defects and whose mothers lived in the northern provinces (Groningen, Drenthe or Friesland) at the time of delivery were eligible for registration.

Cases were ascertained according to EUROCAT’s Central Registry guidelines [26]. EUROCAT-NNL registers foetuses irrespective of gestational age, spontaneous abortions, terminations of pregnancies (foetuses of ≤ 24 weeks’ gestation) following prenatal diagnosis because of a congenital malformation, stillbirths (foetuses of ≥ 24 weeks’ gestation), live births, and children diagnosed before 11 years of age.

Cases are reported by general practitioners, midwives and physicians [27]. Hospital registries are also actively and regularly searched by the EUROCAT-NNL personnel to find eligible children/pregnancies. Various sources including hospital files, obstetric and pathology records are searched for case assessment (type of malformation, chronic diseases and dates of screening procedures). When new information becomes available for an already registered case, the case is updated in the EUROCAT-NNL database until the child reaches the age of 11 years. The paediatric cardiology centre (part of the University Medical Centre Groningen) covers all births in the EUROCAT-NNL registration area and supplies systematic lists with cases and diagnostic details to the registry [28]. For all reported cases, results of genetic tests are downloaded from the genetics department, if these results are available. Abnormal karyotype reports are recorded from prenatal and postnatal samples [28].

Since 1992, parents or guardians are asked to give informed consent for registration of their child and for the use the data for research purposes. The response rate is 80%. Up to 1992, no parental approval was required to register cases.

This study was approved by the Medical Ethical Committee, University Medical Centre Groningen, the Netherlands (number M12.118639).

### Classification

EUROCAT adopted the International Classification of Diseases (ICD-9), with modifications, from the British Paediatric Association for births up to 2001, and the tenth revision (ICD-10) from 2002 onwards [29,30]. The two ICD guides were used to code cases into clinical and anatomical types. There were three clinical types of CLD: (1) isolated CLD, if the case only had one or more limb defects but no other major congenital anomalies; (2) multiple congenital anomalies (MCA), if there was a limb defect combined with at least one major non-limb defect unrelated to a syndrome; or (3) CLD as part of a genetic disorder or syndrome (recognised conditions). There were four anatomical categories: polydactyly (ICD10-Q69 and ICD9-7550), syndactyly (ICD10-Q70 and ICD9-7551, 7550.4), reduction defects (transverse, longitudinal, intercalary and central) (ICD10 Q71-73 and ICD9 7552-7554), and “other CLD” (ICD10-Q74, ICD9-7555, 7556, 7558). Split hand (ICD9 7555.11-7555.14) and split foot (ICD9 7556.12-7556.15) were considered central reduction defects since they are coded as reduction defects in ICD10. Proximal femoral focal deficiency (ICD10-Q72.4, ICD9-7553.80) was classified as an intercalary reduction defect of the lower limb. The “other CLD” classification included limb anomalies like Sprengel’s and Madelung’s deformity, macrodactyly, radioulnar synostosis, hemihypertrophy, limb undergrowth, and arthrogryposis multiplex congenita.

### Study population

All children and foetuses with CLD, diagnosed before or after birth, were included in this study. Children with only minor CLD, such as clinodactyly, camptodactyly, brachydactyly of the fourth and fifth fingers, trigger finger, syndactyly of the second and third toes, sandal gap, and short big toe were not included, because EUROCAT does not register minor CLD. We also excluded malpositions, club foot and dislocation/dysplasia of hips or knees (dislocation of patella) from our analysis because these musculoskeletal anomalies are common birth defects and their inclusion in the calculations of total birth prevalence for CLD would have given an inflated birth prevalence. The ICD-codes were thoroughly checked against the descriptions of CLD and rectified if necessary.

To gain insight into CLD trends, we included children with isolated CLD and MCA (non-syndromic CLD). Because the registration method changed in 1992 with the introduction of informed consent, the trend analysis was conducted for the period 1992–2010. Localisation of the CLD (left/right side) was only studied in live births with isolated CLD or MCA, because of possible lack of information on localisation in stillbirths and spontaneous abortions or termination of pregnancies. Syndromic CLD were excluded from the laterality analysis because they have characteristic patterns [31]. We determined the most frequent anomalies in other organ-systems that occurred in combination with CLD (MCA cases). A clinical geneticist reviewed the cases that were suspected of having monogenic or genetic causality based on the description of the CLD, associated anomalies, and/or family history.

### Statistical analyses

BPs were determined by dividing the number of affected cases by the total number of births (live births, stillbirths, spontaneous abortions/termination of pregnancies) in the EUROCAT-NNL region. To visualise trends, a three-year moving average prevalence was calculated. The χ^2^ test for trend was used to analyse changes over time in BP and to determine whether a type of CLD was preferentially associated with a congenital anomaly affecting another organ system. Only MCA cases that had one type of CLD were included in this analysis. The association between the number of CLD and the number of anomalies in other organ-systems was tested for trend using the χ^2^ test. If χ^2^ assumptions (expected cell counts) were not met, the exact method was used.

Two-tailed values of *P* < 0.05 were considered statistically significant. PASW Statistics 18.0 for Windows (SPSS Inc., 2009, Chicago, IL, http://www.spss.com) was used for the analyses.

## Results

### Birth prevalence and study population

From 1981–2010, 1,048 cases with CLD were recorded among 497,751 births in the northern Netherlands, yielding a BP of 21.1/10,000 (Table 1). The prevalence for transverse reduction defects was 3.9/10,000 births and for longitudinal reduction defects 2.4/10,000 (equal rates for preaxial and postaxial: 1.3/10,000).

**Table 1:** Total birth prevalence per type of congenital limb defects for the period 1981–2010 in the northern Netherlands

**CLD**	**Isolated CLD, *n***	**Multiple congenital anomalies, *n***	**CLD is part of a recognised condition, *n***	**Total, *n***	**Prevalence per 10,000**
Polydactyly	291	45	83	419	8.4
Upper limb	228	38	63^a^	329	6.6
Preaxial	73	13	20	106	2.1
Postaxial	141	21	39	201	4.0
NOS	14	4	6	24	0.5
Lower limb	75	9	32	116	2.3
Preaxial	12	1	8	21	0.4
Postaxial	59	7	19	85	1.7
NOS	4	1	5	10	0.2
NOS	4	0	2	6	0.1
Upper and lower limb	16	2	14	32	0.6
Reduction defects	180	42	120	342	6.9
Upper limb	128	28	94	250	5.0
Transverse	89	17	39	145	2.9
Longitudinal	21^b^	9	51	81	1.6
Preaxial	12	7	33	52	1.0
Postaxial	10	2	18	30	0.6
Intercalary	3	1	5	9	0.2
Central	16	1	4	21	0.4
Multiple	1	0	5	6	0.1
Lower limb	66	25	58	149	3.0
Transverse	29	15	31	75	1.5
Longitudinal	23^c^	5	24	52	1.0
Preaxial	4	3	9	16	0.3
Postaxial	21	2	16	39	0.8
Intercalary	7	5	10	22	0.4
Central	13	1	2	16	0.3
Multiple	7	2	8^d^	17	0.3
NOS	1	1	0	2	0.04
NOS	0	0	1	1	0.02
Upper and lower limb	14	11	33	58	1.2
Syndactyly	126	29	77	232	4.7
Upper limb	77	17	46	140	2.8
Lower limb	59	12	46	117	2.4
NOS	0	1	0	1	0.02
Upper and lower limb	10	1	15	26	0.5
Other CLD^e^	41	34	88	163	3.3
Upper limb	18	14	66	98	2.0
Lower limb	25	22	53	100	2.0
NOS	0	1	0	1	0.02
Upper and lower limb	2	3	31	36	0.7
Multiple CLD^f^	38^g^	14^g^	49^g^	101	2.0
Total no. of cases	598	135	315	1048	21.1
Total no. of live births	587	77	159	823	16.5

Total number of births for the period 1981–2010: n = 497,751.

Abbreviations: CLD–congenital limb defects, NOS–not otherwise specified, *n*–number of children with CLD.

^a^Two children had preaxial and postaxial polydactyly of upper limbs.

^b^One child had preaxial and postaxial longitudinal reduction defects of upper limbs.

^c^Two children had preaxial and postaxial longitudinal reduction defects of lower limbs.

^d^One child had transversal, longitudinal and intercalary reduction defects of lower limbs.

^e^The category consisted of CLD like arthrogryposis (n = 40), hemihypertrophy (n = 25), contractures of elbows/knees/fingers (n = 19), undergrowth of limbs (n = 9), radio-ulnar synostosis (n = 5), macrodactyly (n = 4).

^f^Category containing cases with several CLD included in the study.

^g^Children in the group with isolated (n = 2), multiple congenital anomalies (n = 1), and CLD as part of a recognised condition (n = 4) had three types of CLD, whereas the rest of the children with multiple limb defects had two types of CLD.

**Figure 1:**
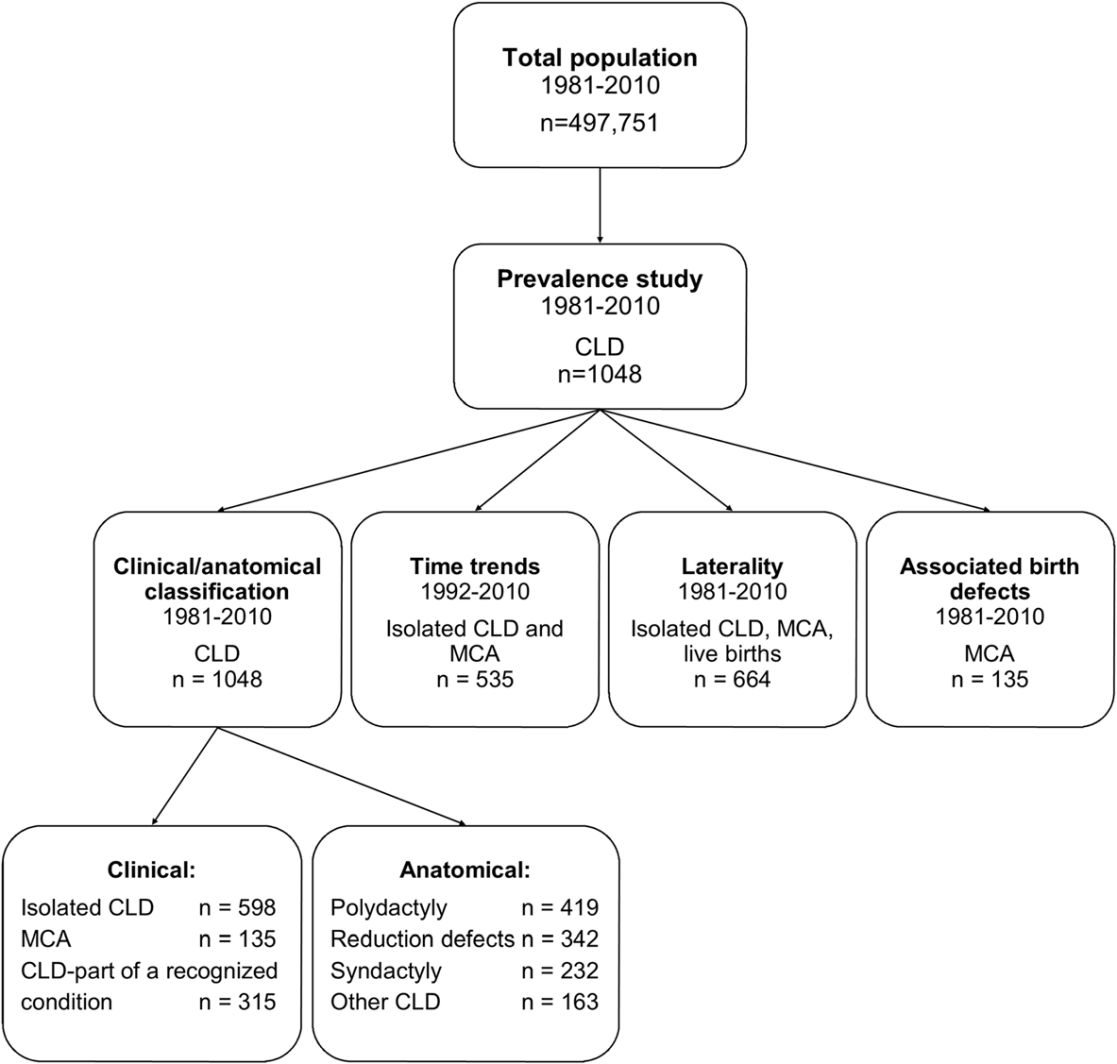
**Overview of population included in the northern Netherlands study.** Abbreviations and notations: CLD–congenital limb defects, MCA–multiple congenital anomalies, n–number of children with CLD.

Of the 1,048 cases, 823 (79%) were live-born children; 181 (17%) were spontaneous abortions, stillbirths or infants who died shortly after birth; and 44 (4%) were termination of pregnancies. Of all 1,048 cases, 578 (55%) were males and 4 were of undetermined gender. More males (455/823; 55%) were also registered in the live births. An overview of the data is given in Figure 1.

### Classification

There were 598 (57%) isolated CLD cases and 135 (13%) MCA cases (Table 1). The remaining 315 (30% of total) cases had a recognised condition, which included 96 chromosomal defects (31%), 103 monogenic defects (33%), 9 deletions (3%), and 107 other recognised conditions (34%) (Table 2). Trisomy 13 (n = 29; 30% of chromosomal defects) and trisomy 18 (n = 24; 25%) were found most often in the cases with chromosomal abnormalities. Cases with trisomy 13 most often had postaxial polydactyly of an upper limb (n = 19), while the monogenic abnormalities contained, for example, cases with arthrogryposis with a known gene (n = 12), Greig syndrome (n = 10), and Holt-Oram syndrome (n = 7). Other recognised conditions were mainly amniotic bands (n = 27, 25%), of which most of the cases had transverse reduction defects (14 upper limb, 10 lower limb) and syndactyly (9 upper limb, 4 lower limb), arthrogryposis (n = 14, 13%) and VATER/VACTERL association (vertebral defects, anal atresia, cardiac anomalies, tracheo-oesophageal fistula with oesophageal atresia, renal dysplasia, limb defects (n = 13, 12%)).

**Table 2:** Description of the recognised conditions with congenital limb defects (CLD)

**Recognised condition**	**CLD, *n***	**Type of CLD and the number of cases**
Chromosomal	96	
Trisomy 13, Patau	29	Polydactyly: Preax. LL = 1, Postax. UL = 19 and LL = 6, NOS UL = 4 and LL = 3
		Syndactyly LL = 1
Trisomy 18, Edwards	24	Polydactyly: Preax. UL = 2, Postax. UL = 3
		Reduction: Transv. UL = 1 and LL = 1, Preax. UL = 7 (radius aplasia, thumb aplasia/hypoplasia) and LL = 1, Postax. UL = 2
		Syndactyly: UL = 2, LL = 5
		Other CLD: UL = 3, LL = 3
Triploidy 69	11	Polydactyly Preax. UL = 1
		Syndactyly: UL = 7, LL = 8
		Other CLD LL = 1 (shortening toes)
Trisomy 21, Down	6	Polydactyly Preax. LL = 1
		Syndactyly: UL = 2, LL = 3
Trisomy 13, translocation	3	Polydactyly: Postax. UL = 2 and LL = 1, NOS UL = 1 and LL = 1
Unlisted chromosomal anomaly^a^	23	
Monogenenic	103	
Arthrogryposis with a known gene	12	Other CLD: UL = 12 and LL = 8 (joint contractions)
Greig syndrome	10	Polydactyly: Preax. UL = 3 and LL = 7, Postax. UL = 4 and LL = 1
		Syndactyly UL = 2, LL = 6
Holt-Oram syndrome	7	Polydactyly Preax. UL = 3 (triphalangeal thumb)
		Reduction Preax. UL = 4 (radius aplasia/dysplasia)
Apert syndrome	5	Polydatyly Preax. UL = 1
		Syndactyly: UL = 4 (3 digits II-V, 1 all digits), LL = 3 (all digits)
		Other CLD = 2
Cornelia de Lange syndrome	5	Reduction: Transv. UL = 4 (bilateral), Postax. LL = 1, Central UL = 1 (split hand)
		Syndactyly LL = 1
		Other UL = 3 (all monodactyly)
Bardet-Biedl syndrome	4	Polydactyly: Postax. UL = 3 (bilateral) and LL = 3 (2 bilateral)
		Reduction Transv. LL = 1
Thanatophoric dysplasia/dwarfism	3	Reduction: Transv. UL = 1 and LL = 1, Intercalary UL = 2 and LL = 2
Meckel-Gruber syndrome	3	Polydactyly: Postax. UL = 2 (1 bilateral) and LL = 2 (bilateral)
		Syndactyly: LL = 1 (bilateral)
Peters plus syndrome	3	Reduction: Transv. UL = 2 (short UL) and LL = 1 (short LL), Intercalary LL = 1 (reduction of femur bilateral);
Unlisted monogenic^a^ anomaly	51	
Deletions	9	Polydactyly: Preax. UL = 1, Postax. UL = 1
		Reduction: Transv. UL = 1 and LL = 1, Intercalary UL = 1 and LL = 1, Central UL = 1 (split hand) and LL = 1 (split foot)
		Syndactyly: UL = 1, LL = 3
Other recognised conditions	107	
Amniotic bands	27	Reduction: Transv. UL = 14 and LL = 10, Preax. UL = 2 and LL = 6, Postax. UL = 5, Intercalary UL = 1
		Syndactyly: UL = 9, LL = 4
		Other CLD: UL = 4, LL = 5 (constriction bands)
Caudal regression syndrome	4	Reduction: Transv. UL = 1 and LL = 1, Preax. UL = 1 (atresia radius and thumb), Postax. LL = 1
		Syndactyly UL = 1
Acardiacus	3	Reduction: Transv. UL = 1 and LL = 1, Postax. UL = 1 and LL = 1, NOS = 1
		Syndactyly LL = 1
Femoral facial syndrome	3	Reduction Intercalary = 1 (femoral hypoplasia)
		Syndactyly LL = 2
		Other CLD = 1 (contractures elbows and knees)
Limb–body-wall complex	6	Reduction: Transv. UL = 1 and LL = 3 (right side), Intercalary UL = 1, Postax. LL = 2
		Other CLD LL = 1
Oculo-auriculo-vertebral spectrum	4	Polydactyly Preax. UL = 1
		Reduction Transv. UL = 1
		Other CLD: UL = 1 (Sprengel deformity), LL = 1 (hemihypertophy)
VATER/VACTERL association	13	Polydactyly: Preax. UL = 2, Postax. LL = 1
		Reduction: Transv. UL = 1 and LL = 1, Preax. UL = 8 (radius aplasia with or without thumb agenesis/hypoplasia), Central UL = 1 (split hand)
		Other CLD LL = 1 (flexion-extension deformity)
Poland syndrome	4	Reduction: Preax. UL = 2 (radius aplasia/dysplasia and thumb aplasia), Intercalary UL = 1
		Syndactyly UL = 3
Foetal valproate syndrome	3	Polydactyly Preax. UL = 1
		Reduction Preax. UL = 2 (radius aplasia)
		Syndactyly UL = 1
Arthrogryposis multiplex congenita^b^	14	Other CLD: UL = 14 (joints contractures), LL = 11 (joints contractures)
Femur-Fibula-Ulna complex	9	Reduction: Transv. LL = 1, Preax. LL = 1, Postax. UL = 5 (ulna hypoplasia, missing fingers) and LL = 8 (fibula aplasia, missing toes), Intercalary UL = 1 and LL = 4 (femur hypoplasia)
		Syndactyly: UL = 3, LL = 2
		Other CLD UL = 1
Klippel-Trenaunay-Weber syndrome	5	Other CLD: Hypertrophy UL = 3 (entire upper limb = 2, macrodactyly = 1) and LL = 2 (entire lower limb)
Unlisted other recognised conditions^a^	12	
Total	315	

Abbreviations: CLD–congenital limb defects, UL–upper limb, LL–lower limb, Transv.–transversal, Preax.–preaxial, Postax.–postaxial, *n*–number of children with CLD.

^a^Recognised conditions occurring in less than 3 cases are not listed in the table.

^b^Unknown gene.

In this study, termination of pregnancy was performed in 44 cases. Isolated CLD occurred in 2 of the 44 cases (split hands and feet; mixed reduction defects of the lower limb), MCA in 15 (CLD with one or more other major non-CLD defects: CNS and neural tube defects (n = 5), urinary (n = 5), digestive system (n = 4), cardiovascular (n = 3), respiratory system (n = 3)), and recognized conditions in 27 (chromosomal (n = 14), other recognized condition (n = 7), and monogenic (n = 6)).

### Trend analyses for the period 1992–2010

**Figure 2:**
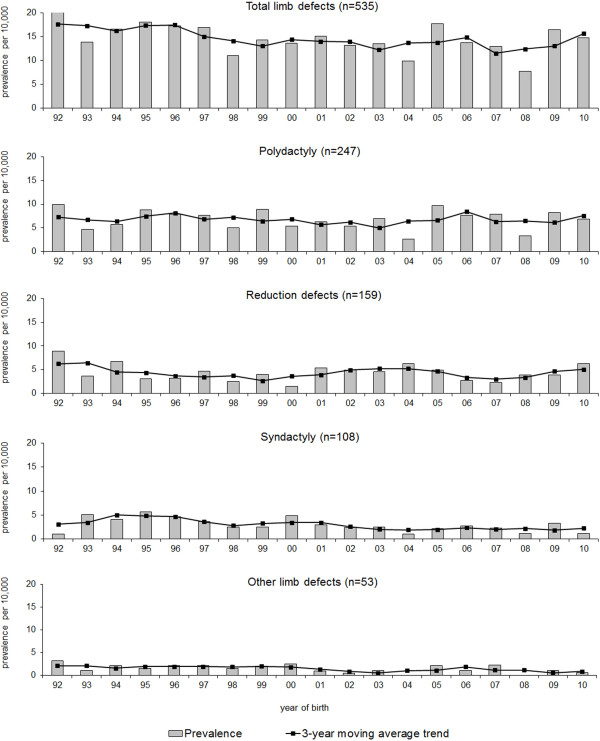
**Time trends for non-syndromic congenital limb defects (isolated and MCA) for the period 1992–2010.** MCA–multiple congenital anomalies. Total limb defects: *P* for trend, 0.023; Polydactyly: *P* for trend, 0.574; Reduction defects: *P* for trend, 0.381; Syndactyly: *P* for trend, 0.009; Other congenital limb defects (CLD): *P* for trend, 0.028.

A significant decrease of the BP rate over time was found for non-syndromic CLD as a group and for non-syndromic syndactyly in particular (CLD: χ^2^ = 5.2, *P* = 0.023, syndactyly: χ^2^ = 6.8, *P* = 0.009) (Figure 2). The decrease in non-syndromic syndactyly from 5.2/10,000 to 1.1/10.000 births was also found to be responsible for the decrease of non-syndromic CLD as a total group (CLD syndactyly excluded: χ^2^ = 1.5, *P* = 0.215). A significant decrease was also noticed in the heterogeneous group of “other CLD” (χ^2^ = 4.8, *P* = 0.028). When we included recognised conditions in the analysis, no trend was identified for syndactyly (χ^2^ = 1.5, *P* = 0.218), but there was a significant decreasing trend present for CLD as a group (χ^2^ = 9.3, *P* = 0.002).

### Localisation and laterality of limb defects

Upper limbs were more often affected than lower limbs (upper:lower = 2:1). Upper versus lower limb ratios were 3:1 in polydactyly, 1.5:1 in syndactyly, and 2:1 in reduction defects (Table 3). Limb defects were more often left-sided (left:right = 1.2:1).

**Table 3:** **Description of laterality in live-born children with a limb defect**^**a**^

	**Right,**	**Left,**	**Bilateral,**	**Total of sites,**^**b**^
	***N *** **(%)**	***N *** **(%)**	***N *** **(%)**	***N *** **(%)**
Polydactyly (n = 322)	90 (13.6)	98 (14.8)	118 (17.8)	306 (46.1)
Upper limb	65 (9.8)	72 (10.8)	95 (14.3)	232 (34.9)
Lower limb	25 (3.8)	26 (3.9)	23 (3.5)	74 (11.1)
Syndactyly (n = 140)	40 (6.0)	49 (7.4)	44 (6.6)	133 (20.0)
Upper limb	24 (3.6)	29 (4.4)	26 (3.9)	79 (11.9)
Lower limb	16 (2.4)	20 (3.0)	18 (2.7)	54 (8.1)
Reduction defects (n = 193)	78 (11.7)	88 (13.3)	34 (5.1)	200 (30.1)
Upper limb	52 (7.8)	67 (10.1)	14 (2.1)	133 (20.0)
Transverse	38 (5.7)	49 (7.4)	7 (1.1)	94 (14.2)
Longitudinal	8 (1.2)	9 (1.4)	6 (0.9)	23 (3.5)
Intercalary	0	2 (0.3)	0	2 (0.3)
Central	6 (0.9)	7 (1.1)	1 (0.2)	14 (2.1)
Lower limb	26 (3.9)	21 (3.2)	20 (3.0)	67 (10.1)
Transverse	10 (1.5)	10 (1.5)	10 (1.5)	30 (4.5)
Longitudinal	10 (1.5)	9 (1.4)	3 (0.5)	22 (3.3)
Intercalary	3 (0.5)	1 (0.2)	0	4 (0.6)
Central	3 (0.5)	1 (0.2)	7 (1.1)	11 (1.7)
Other CLD (n = 58)	18 (2.7)	32 (4.8)	9 (1.4)	59 (8.9)
Upper limb	5 (0.8)	14 (2.1)	4 (0.6)	23 (3.5)
Lower limb	13 (2.0)	18 (2.7)	5 (0.8)	36 (5.4)
Total CLD (n = 664)	226 (34.0)	267 (40.2)	205 (30.9)	698 (105.1)
Upper limb	146 (22.0)	182 (27.4)	139 (20.9)	467 (70.3)
Lower limb	80 (12.0)	85 (12.8)	66 (9.9)	231 (34.8)

Abbreviations and notations: CLD–congenital limb defects, *N*–number of sites.

Percentages are calculated from the total number of children with isolated CLD and multiple congenital anomalies (n = 664). In 58 infants localisation was unknown.

^a^Included only live births with isolated and multiple congenital defects (including a limb defect) because of 1) lack of information on stillbirths and abortions, and 2) genetic abnormalities or syndromes have characteristic patterns [31].

^b^Number of sites exceeds the number of children due to multiple CLD in some cases.

### Associated anomalies

CLD were most common with cardiovascular anomalies (Table 4): 19 ventricular septal defects (of which 8 cases had polydactyly), 7 atrial septal defects, 6 tetralogy of Fallot, and 4 coarctation of the aorta. Urinary tract anomalies were also frequent: 12 bi- or unilateral renal agenesis (of which 7 cases had reduction defects), 5 hydronephrosis, and 5 cystic kidney. Anomalies of the central nervous system were found in fewer cases with CLD: 10 cases of hydrocephaly (of which 5 had syndactyly) and 4 of microcephaly. More males were affected in cases with genital anomalies (n = 17, uniformly spread between CLD) of which most were hypospadias (n = 14).

**Table 4:** **Anomalies in other organ systems occurring with congenital limb defects**^**a**^

**Anomalies**	**Polydactyly**			**Syndactyly**	**Total reduction defects**					**Other CLD**	**Total CLD**^**c**^
	**(n = 45)**			**(n = 29)**	**(n = 42)**					**(n = 34)**	**(n = 135)**
	**Total**^**b**^**, *n *** **(%)**	**Preax**	**Postax**	***n *** **(%)**	**Total**^**b**^**, *n *** **(%)**	**Transv**	**Preax**	**Postax**	**Intercal**	**Total, *n *** **(%)**	***n *** **(%)**
CNS and neural tube defects	6 (4.4)	1	4	8 (5.9)	10 (7.4)	7	5	0	0	6 (4.4)	27 (20.0)
Hydrocephaly	1 (0.7)	0	0	5 (3.7)	3 (2.2)	2	2	0	0	3 (2.2)	10 (7.4)
Microcephaly	0	0	0	1 (0.7)	4 (3.0)	3	2	0	0	0	4 (3.0)
Eye	0	0	0	5 (3.7)	2 (1.5)	1	0	1	0	3 (2.2)	9 (6.7)
Ear	2 (1.5)	1	1	1 (0.7)	1 (0.7)	1	0	0	0	1 (0.7)	4 (3.0)
Cardiovascular	19 (14.1)	9	9	7 (5.2)	16 (11.9)	9	6	1	2	17 (12.6)	50 (37.0)
Tetralogy of Fallot	2 (1.5)	2	0	0	2 (1.5)	2	0	0	0	2 (1.5)	6 (4.44)
Atrium septum defects	1 (0.7)	1	0	1 (0.7)	3 (2.2)	2	1	0	0	3 (2.2)	7 (5.2)
Ventricular septum defects	8 (5.9)	4	3	4 (3.0)	6 (4.4)	2	3	1	2	6 (4.4	19 (14.1)
Coarctation aortae	2 (1.5)	0	2	0	1 (0.7)	0	1	0	0	1 (0.7)	4 (3.0)
Respiratory	8 (5.9)	2	4	3 (2.2)	5 (3.7)	2	3	0	0	6 (4.4)	19 (14.1)
Choanal atresia	3 (2.2)	0	2	0	0	0	0	0	0	0	3 (2.2)
Lung hypoplasia	1 (0.7)	0	0	1 (0.7)	0	0	0	0	0	4 (3.0)	5 (3.7)
Clefts	7 (5.2)	2	4	5 (3.7)	3 (2.2)	1	2	0	0	2 (1.5)	15 (11.1)
Cleft palate	3 (2,2)	0	3	3 (2.2)	2 (1.5)	1	1	0	0	1 (0.7)	8 (5.9)
Cleft lip	2 (1.5)	0	1	0	1 (0.7)	0	1	0	0	0	3 (2.2)
Digestive	8 (5.9)	1	6	3 (2.2)	14 (10.4)	8	5	1	0	3 (2.2)	25 (18.5)
Malformations of oesophagus	0	0	0	0	4 (3.0)	2	0	0	0	0	4 (3.0)
Atresia/stenosis large intestine	2 (1.5)	0	2	0	5 (3.7)	3	2	0	0	0	7 (5.2)
Anorectal atresia	2 (1.5)	0	2	0	5 (3.7)	3	2	0	0	0	7 (5.2)
Genital	6 (4.4)	0	6	3 (2.2)	10 (7.4)	6	4	0	0	4 (3.0)	23 (17.0)
Female	1 (0.7)	0	1	0	4 (3.0)	1	2	0	0	0	5 (3.7)
Male	5 (3.7)	0	5	3 (2.2)	5 (3.7)	3	1	0	0	4 (3.0)	17 (12.6)
Hypospadias	4 (3.0)	0	4	3 (2.2)	3 (2.2)	2	1	0	0	2 (1.5)	12 (8.9)
Urinary	6 (4.4)	1	3	10 (7.4)	13 (9.6)	6	6	1	1	8 (5.9)	34 (25.2)
Renal agenesis (uni/bilateral)	3 (2.2)	1	1	1 (0.7)	7 (5.2)	5	3	1	0	1 (0.7)	12 (8.9)
Cystic kidney	0	0	0	1 (0.7)	3 (2.2)	1	2	0	1	1 (0.7)	5 (3.7)
Potter sequence	0	0	0	1 (0.7)	2 (1.5)	1	2	0	0	1 (0.7)	4 (3.0)
Hydronephrosis	1 (0.7)	0	0	2 (1.5)	0	0	0	0	0	2 (1.5)	5 (3.7)
Horseshoe kidney	0	0	0	1 (0.7)	1 (0.7)	0	0	0	0	3 (2.2)	4 (3.0)
Abdominal wall defects	2 (1.5)	0	1	0	1 (0.7)	1	0	0	0	2 (1.5)	5 (3.7)
Omphalocele	1 (0.7)	0	1	0	1 (0.7)	1	0	0	0	2 (1.5)	4 (3.0)
Other	4 (3.0)	2	2	3 (2.2)	2 (1.5)	2	0	0	0	4 (3.0)	12 (8.9)

Abbreviations and notations: CLD–congenital limb defects, CNS–central nervous system, Transv–transversal, Preax–preaxial, Postax–postaxial, Intercal–intercalary, *n*–number of children with CLD.

^a^Only cases with multiple congenital anomalies (including a limb defect) were included because genetic abnormalities or syndromes have particular associations [17]. General categories and examples of anomalies occurring with CLD were given.

^b^Numbers do not always add up due to multiple CLD in some cases or due to lack of information on subcategories of CLD (e.g. preaxial, postaxial). Percentages are calculated from the total number of children with multiple congenital anomalies and CLD (n = 135).

^c^In addition to one or several major non-limb defects, 24 cases (17.8%) also had a malposition, or a clubfoot, or hip dysplasia/dislocation (polydactyly n = 1, syndactyly n = 8, reduction defects n = 9, other CLD n = 12).

We only found a significant association between digestive anomalies and CLD (*P* = 0.016). Reduction defects were more likely to occur in combination with digestive tract anomalies, whereas syndactyly and “other CLD” were less likely to occur with such anomalies (Table 5). We found no significant association between the number of CLD and the number of anomalies in other organ-systems (χ^2^ = 3.4, df 1, linear-by-linear association *P* = 0.067).

**Table 5:** **Associated anomalies with congenital limb defects**^**a**^

**Anomalies**		**Polydactyly**	**Reduction defects**	**Syndactyly**	**Other CLD**	**Total**		
		***n *** **(%)**	***n *** **(%)**	***n *** **(%)**	***n *** **(%)**	***n *** **(%)**	**χ**^**2**^	***P***
Cardiovascular	yes	15 (45.5)	9 (27.3)	1 (3.0)	8 (24.2)	33 (100)	1.98	0.160
	no	25 (35.7)	21 (30.0)	14 (20.0)	10 (14.3)	70 (100)		
Urinary anomalies	yes	5 (21.7)	9 (39.1)	5 (21.7)	4 (17.4)	23 (100)	1.85	0.194^#^
	no	35 (43.8)	21 (26.3)	10 (12.5)	14 (17.5)	80 (100)		
CNS and neural tube defects	yes	6 (31.6)	7 (36.8)	4 (21.1)	2 (10.5)	19 (100)	1.63	0.246^#^
	no	34 (40.5)	23 (27.4)	11 (13.1)	16 (19.0)	84 (100)		
Genital	yes	6 (31.6)	8 (42.1)	2 (10.5)	3 (15.8)	19 (100)	1.09	0.354^#^
	no	34 (40.5)	22 (26.2)	13 (15.5)	15 (17.9)	84 (100)		
Digestive	yes	7 (35.0)	11 (55.0)	1 (5.0)	1 (5.0)	20 (100)	6.23	0.016^#*^
	no	33 (39.8)	19 (22.9)	14 (16.9)	17 (20.5)	83 (100)		
Respiratory	yes	7 (50.0)	4 (28.6)	0	3 (21.4)	14 (100)	0.52	0.514^#^
	no	33 (37.1)	26 (29.2)	15 (16.9)	15 (16.9)	89 (100)		
Clefts (palate, lip)	yes	6 (54.5)	2 (18.2)	2 (18.2)	1 (9.1)	11 (100)	0.10	0.774^#^
	no	34 (37.0)	28 (30.4)	13 (14.1)	17 (18.5)	92 (100)		
Eye	yes	0	1 (20.0)	2 (40.0)	2 (40.0)	5 (100)	0.10	0.838^#^
	no	40 (40.8)	29 (29.6)	13 (13.3)	16 (16.3)	98 (100)		
Ear	yes	2 (66.7)	1 (33.3)	0	0	3 (100)	0.03	1.000^#^
	no	38 (38.0)	29 (29.0)	15 (15.0)	18 (18.0)	100 (100)		
Total cases per CLD type		40 (38.8)	30 (29.1)	15 (14.6)	18 (17.5)	103 (100)		

Abbreviations and notations: CLD–congenital limb defects, *n*–number of children with CLD, CNS–central nervous system, χ^2^–test value, *P*–value showing the significance of association of anomalies with limb defect.

^a^Only MCA cases with multiple congenital anomalies that had one type of CLD were included in this analysis; cases with a CLD and a malposition, or hip dysplasia/dislocation, or clubfoot were excluded from the analysis.

^*^Significant *P* value.

^#^Exact *P* values.

## Discussion

We aimed to describe the epidemiology of CLD in the northern Netherlands. From 1981–2010, the birth prevalence of CLD was 21.1/10,000 births, which falls between the BPs found in two other European registries: Finland (13/10,000 for 1964–1977) and Scotland (30.4/10,000 for 1964–1968) [9,10]. However, there were differences in the inclusion criteria of all three registries.

More recent BPs for CLD are available on the official website of the EUROCAT network of over thirty-seven national registries [32]. These registries include live births, stillbirths and terminations of pregnancies, which allow their BPs for types of CLD to be compared with the Netherlands (NNL). Six registries have reported complete data for the period 1981–2010 (Dublin, Ireland; Odense, Denmark; Paris, France; Hainaut, Belgium; Emilia-Romagna and Tuscany, Italy).

Our BP for polydactyly (8.4/10,000) was close to the figures reported for Emilia-Romagna and Hainaut (8.14 and 8.55; Table 6), but much lower that the BP in Paris (13.8). We found a BP for syndactyly of 4.7/10,000, which is comparable to that reported for Emilia-Romagna (4.5) and Paris (5.1). Our BP for reduction defects (6.9) was close to that reported by Hainaut (7.3; Table 6).

**Table 6:** Birth prevalences per 10,000 births in six EUROCAT registries for the period 1981-2010

**Registry**	**Polydactyly**	**Reduction defects**	**Syndactyly**	**Total of the three CLD**
Ireland - Dublin	7.6	5.7	4.3	17.6
Denmark - Odense	7.8	8.2	6.2	22.2
France - Paris	13.8	8.0	5.1	26.9
Belgium - Hainaut	8.6	7.3	7.6	23.4
Italy - Emilia Romagna	8.1	5.4	4.5	18.0
Italy - Tuscany	7.2	5.2	6.2	18.6
Northern Netherlands^a^	8.8	8.6	5.8	23.2
Northern Netherlands^b^	8.4	6.9	4.7	20.0

^a^Birth prevalences for the northern Netherlands on the EUROCAT website differ from the ones reported in this study due to thorough verification and corrections of miscoding.

^b^Birth prevalences in this study.

The total BP of each EUROCAT registry includes the club foot and hip dysplasia/dislocation, which hampers direct comparison with the total BP determined in our study (21.1/10,000). However, we can compare the summed BPs for polydactyly, syndactyly and reduction defects. Our calculated BP (20/10,000) is similar to those calculated for Emilia-Romagna (18.0), Tuscany (18.6), and Odense (22.2) (Table 6).

A literature review summarized the BPs of reduction defects in different countries and time periods [23]. BPs varied from 3.3 to 8.1 (in 1970) and to 5.0/10,000 in Canada (Alberta, 1966–1975), from 6.6 to 4.8/10,000 in USA (Atlanta, 1968–1993), and even to 10.4/10,000 births in France (1979–1987) [23]. The authors also mentioned that the BPs of reduction defects may have been underestimated in countries that excluded terminations of pregnancies from their registries. The latest advances in prenatal diagnosis are leading to more terminations because of CLD [23]. In our NNL study, the most common type of reduction defects were transverse (3.9/10,000). Some studies reported similar prevalences for this type: France had 4.3/10,000 births for 1979–1987, Italy 2.6/10,000 for 1978–1987, and there was a prevalence of 4.0/10,000 in six combined EUROCAT registries (Strasbourg, Belfast, Emilia Romagna, Odense, Groningen, Basque Country) [13,19,24]. A recent study in the USA found more longitudinal (3.5/10,000 births) than transverse reduction defects (1.9/10,000 births) for the periods 1972–1974 and 1979–2000 [3].

The differences in BP between countries are most likely to be the result of variations in coding methods, ascertainment, notification and inclusion criteria and, until a consistent and compatible system (such as that of EUROCAT) is universally adopted, there will be no way of determining whether BP variation has an environmental or other cause.

### Localisation and laterality of limb defects

We found upper limbs were more commonly affected, which agrees with the literature [9,13,23,33]. Left-sided limb defects prevailed in our study, as in other studies [9,14,24], but others have reported more right-sided defects [16,34] or both sides being equally affected [35]. Longitudinal and multiple reduction defects were more frequently localised on the right side [34].

### Gender distribution

Our male:female ratio for CLD was 1.2:1 in both total births and in live births, compared with a male:female ratio of 1.1:1 for live births in the northern Netherlands (1981–2010) [36]. This male excess has been reported before, but its aetiology remains unexplained [17,19,37].

### Trends

In the northern Netherlands, the BP of non-syndromic syndactyly has shown a significant decrease since 1992. EUROCAT-NNL has a reliable and well-established network that notifies the registry of new cases shortly after birth, ensuring that the parents of almost all children with congenital anomalies in the region are contacted for registration, thus we do not feel many cases will have been missed. In addition, the diagnosis of syndactyly as part of a genetic syndrome is more commonly detected now than in the early 1990s. Excluding cases with genetic syndromes from our time-trend analysis may have influenced our finding of a drop in cases with non-syndromic syndactyly over time. Another reason for the decline might be a change in parental attitude towards giving informed consent for registering syndactyly, which might now be seen as a rather minor anomaly. The introduction of the informed consent procedure in 1992 may also have influenced the number of registrations compared to the period before 1992. In a post-hoc analysis, no other significant trends were found for the period 1981–1991. Finally, not all CLD cases may have been reported yet. By including data from recent years in our study, the decline may be due to the fact that some of the cases still need to be registered. Therefore, we cannot fully rule out that the decline observed is a registration artefact.

### Associated birth defects and recognised conditions

In comparison with polydactyly, syndactyly and “other CLD”, we saw reduction defects more often in combination with congenital anomalies affecting the central nervous system, digestive system (the only significant association in current study, Table 5), genital or urinary system. Associations of reduction defects with these kinds of anomalies have been reported previously [17,19,24]. However, in our study, reduction defects were not the CLD type that occurred most frequently in combination with cardiovascular anomalies, as seen in other studies [17,19,24]. We found polydactyly (equally preaxial and postaxial) occurred most often in combination with cardiovascular anomalies, especially with ventricular septum defects. Another study reported polydactyly occurring mostly with central nervous system anomalies [38]. They also found significant associations of polydactyly with recognised syndromes: trisomy 13, Meckel, and Down syndrome [38]. We only found trisomy 13 occurring often in our cases with postaxial polydactyly of the upper limb.

In our NNL study, amniotic bands were the second most frequently recognised condition after trisomy 13, and they also occurred more often in combination with transversal limb defects and syndactyly, as reported in the literature [34]. We also found syndactyly occurring often with trisomy 18 (mostly syndactyly of lower limbs), Apert, and Poland syndrome, also reported previously [39].

Longitudinal preaxial reduction defects were found to be the most common reduction defects occurring in cases with other congenital anomalies [17,34]. Preaxial defects, for instance, can occur in the VATER association and in many genetic conditions, while transverse defects are usually isolated single defects in a family and may be caused by disruptive events in early pregnancy [17,34]. We also found radius aplasia/dysplasia most frequently in cases with VATER/VACTERL, trisomy 18, and Holt-Oram syndrome.

To our knowledge, there are no genetic predispositions that are specific to the Dutch population that could have influenced the birth prevalences of CLDs in this study. In addition, the tables on the official EUROCAT website did not reveal any particular recognized condition specific for the Netherlands compared to the other six registries (Dublin, Ireland; Odense, Denmark; Paris, France; Hainaut, Belgium; Emilia-Romagna, and Tuscany, Italy) [32]. These observations, and the fact that our birth prevalences of CLD are comparable to those in other EUROCAT registries, imply that our birth prevalences are relatively good estimates of world-wide prevalences. However, studies on larger populations with CLD and with the same inclusion criteria would allow for better generalizability.

### Study strengths and limitations

The EUROCAT-NNL database contains specific and detailed information about cases with birth defects. All the cases were verified and corrected for any miscoding of the CLD. Updated information, if available, was retrieved from the notifying hospital to clarify doubtful coding or diagnosis.

Our data will be of clinical relevance to clinicians treating children with CLD and to their parents, not only the parents of live-born children who were unaware of their child’s defect prior to delivery, but also the parents who know they are expecting a child with a CLD. The latter group is becoming more and more significant with all the possibilities for information now offered by prenatal diagnostics.

A limitation of our study may be the relatively small number of children with MCA included in the analysis for associations. The response rate for the parental consent for the registration of their child is fairly high (80%). Nevertheless, we cannot exclude the fact that underestimation of the true BP of CLD may have occurred. This type of underestimation, due to the voluntary participation of parents or notifying sources, is also likely to be present in other birth defects registries (e.g., Tuscany and Emilia-Romagna, Italy; Paris, France; United Kingdom; Austria; Switzerland; Spain) [28]. A further limitation may be the classification system used. Although there are several classifications of CLD [1,40,41] available, they all pose difficulties. The International Federation of Societies for Surgery of the Hand has adopted the morphological classification of Swanson et al. [40]. However, this classification for CLD has not been accepted by all surgeons [14], and difficulties based on this classification have been reported [20]. Furthermore, Swanson’s classification is a more morphological classification, and does not conform to the aim of our study, which was to provide a descriptive overview of CLD. Stoll et al. [42] proposed a purely descriptive classification of CLD, which complied with the purposes of this article. However, Stoll et al.’s classification did not allow for CLD that were included in the “other CLD” category (e.g. arthrogryposis). We therefore used a classification based on ICD-9 and ICD-10 [29,30].

## Conclusions

We established a prevalence of 21.1 children with CLD per 10,000 births in NNL after a thorough coding and correction procedure, which makes this figure reasonably reliable. The decreasing trend observed in non-syndromic syndactyly in 1992–2010 may be real or the result of a registration artefact. CLD occurred frequently in combination with cardiovascular and urinary tract anomalies, and were significantly associated with digestive-tract anomalies.

## Abbreviations

BPA: British Paediatric Association; BP: Birth prevalences; CLD: Congenital limb defects; EUROCAT: European Surveillance of Congenital Anomalies; ICD: International Classification of Diseases; MCA: Multiple congenital anomalies; SPSS: Statistical Package for the Social Sciences.

## Competing interests

The authors declare that they have no competing interests.

## Authors’ contributions

EV, CvdS, HRM, and HdW participated in the design of the study. EV and HdW prepared the database. EV, CvdS, HdW, AvE, and JB participated in the assessment of diagnoses and correction of the incorrect codes describing the birth defects. EV, HRM, PUD, and HdW participated in the analysis of the data. EV has written the paper. CvdS, AvE, JB, PUD, HRM, and HdW contributed with critical revision of manuscript for important intellectual content. All authors have approved this manuscript for submission.

## Pre-publication history

The pre-publication history for this paper can be accessed here:

http://www.biomedcentral.com/1471-2474/14/323/prepub
